# SARS-CoV-2 Spike Protein Vaccine-Induced Immune Imprinting Reduces Nucleocapsid Protein Antibody Response in SARS-CoV-2 Infection

**DOI:** 10.1155/2022/8287087

**Published:** 2022-07-29

**Authors:** Juan F. Delgado, Mónica Vidal-Pla, M. Carmen Moya, Mateu Espasa, Antonio Casabella, Manel Seda, Joan Calvet, Jordi Gratacós, Rosa M. Serrano, Pilar Peña

**Affiliations:** ^1^Immunology Unit, Laboratory Service, Parc Taulí Hospital Universitari, Institut d'Investigació i Innovació Parc Taulí (I3PT), Universitat Autònoma de Barcelona, Departament de Medicina, Sabadell, Spain; ^2^Occupational Health Department, Parc Taulí Hospital Universitari, Institut d'Investigació i Innovació Parc Taulí (I3PT), Universitat Autònoma de Barcelona, Sabadell, Spain; ^3^Microbiology Unit, Laboratory Service, Parc Taulí Hospital Universitari, Institut d'Investigació i Innovació Parc Taulí (I3PT), Universitat Autònoma de Barcelona, Sabadell, Spain; ^4^Rheumatology Service, Parc Taulí Hospital Universitari, Institut d'Investigació i Innovació Parc Taulí (I3PT), Universitat Autònoma de Barcelona, Departament de Medicina, Sabadell, Spain

## Abstract

Immune imprinting or original antigenic sin (OAS) is the process by which the humoral memory response to an antigen can inhibit the response to new epitopes of that antigen originating from a second encounter with the pathogen. Given the situation of the COVID-19 pandemic, multiple vaccines have been developed against SARS-CoV-2 infection. These vaccines are directed to the spike protein (S protein) of the original variant of Wuhan D614G. Vaccine memory immune response against S protein in noninfected subjects could inhibit, through the OAS mechanism, the response to new epitopes of SARS-CoV-2 after infection. The present study analyzes whether the memory antibody B cell response generated by mRNA vaccines against S protein can inhibit the primary antibody immune response to other SARS-CoV-2 antigens, such as nucleocapsid protein (N protein). SARS-CoV-2 primary infection in vaccinated healthcare workers (HCWs) produced significantly lower titers of anti-N antibodies than that in nonvaccinated HCWs: 5.7 (IQR 2.3-15.2) versus 12.2 (IQR 4.2-32.0), respectively (*p* = 0.005). Therefore, spike protein vaccine-induced immune imprinting (original antigenic sin) reduces N protein antibody response.

## 1. Introduction

Immune imprinting or original antigenic sin (OAS) refers to the immune system's preference to activate existing memory cells when an individual encounters a new but closely related antigen rather than stimulating de novo responses to the new epitopes. This mechanism has been reported in mRNA viruses, such as influenza, dengue, and HIV [1, 2]. According to OAS, when a virus infects an organism, the individual develops a primary immune response to virus antigens that activates innate and adaptive immunity leading to the production of memory T and B cells. In a second entry of the same virus, the organism develops a secondary immune response that is of great intensity and affinity and faster than the primary response. This secondary response can even inhibit a primary response against new epitope antigens if the virus partially modifies its antigen [1–4]. The goal of vaccination is to develop a strong and efficient response from T and B cells that leads to a mild infection. A problem arises when the pathogen acquires the ability to slightly change its antigens, and due to the OAS mechanism, it could inhibit the primary response against new antigens, reducing the immune system's ability to neutralize the pathogen and, therefore, leading to more severe disease [1–4].

Given the situation of the COVID-19 pandemic, multiple vaccines have been developed against SARS-CoV-2 infection. The first vaccines approved by the American and European drug agencies have been the mRNA vaccines. These vaccines are directed to the spike protein (S protein) of the original variant of Wuhan D614G [5, 6]. In the evolution of the pandemic, new variants with mutations in the S protein have emerged. These mutations in the S protein could induce an OAS process in which the memory response against conserved areas of the S protein inhibits the response to new epitopes generated by these mutations [1–6].

The present study analyzes whether the memory antibody B cell response generated by mRNA vaccines against S protein can inhibit the primary antibody immune response to other SARS-CoV-2 antigens, such as nucleocapsid protein (N protein). Therefore, antibody response against N protein in both healthcare workers (HCWs) infected by SARS-CoV-2 after vaccination and nonvaccinated HCWs was analyzed and compared.

## 2. Results

The demographic and analytical characteristics of the HCWs included in the study were similar between vaccinated and nonvaccinated groups, except for antibody titers against N protein after infection (Table 1). All HCWs in the vaccinated group after infection have increased antibody titers against S protein (>34 fold change, *p* < 0.001). The real magnitude of this increase cannot be appreciated since there is an upper limit of 25,000 BAU/mL due to the maximum automatic sample dilution by the Cobas e 801 analyzer. This S protein antibody response we observed was mainly due to the memory antibody response induced by vaccination. Subsequently, we have analyzed the N protein antibody response in HCWs from the vaccinated group and from the nonvaccinated group to evaluate the influence of the S protein memory antibody response in another SARS-CoV-2 antigen. To evaluate this response, we have analyzed the different registered variables shown in Table 1 as possible confounding factors, studying the influence of each of these variables using linear regression models. Coefficient variation applying the variables changes by less than 5%, given the homogeneous distribution of the different variables in both groups (Table 1). Therefore, S protein vaccine-induced immune imprinting is an independent variable when analyzing its effect on the antibody response to N protein. N protein response is greater in the HCWs from the nonvaccinated group compared to the vaccinated group: 12.2 (IQR 4.2-32.0) versus 5.7 (IQR 2.3-15.2), respectively. This 2.1 fold change between groups was statistically significant (*p* = 0.005) (Table 1).

## 3. Discussion

OAS is the process by which the humoral memory response to an antigen can inhibit the response to new epitopes of that antigen originating from a second encounter with the pathogen [7]. mRNA vaccines are administered intramuscularly, and the mRNA encoding for protein S enters into different cells, including antigen-presenting cells. These cells circulate to the lymph node and present S protein epitopes to cytotoxic T cells and helper T cells (Th). A subset of Th cells that recognize S protein epitopes will differentiate into follicular Th cells (Thf) that will traffic to the germinal center. These Thf cells participate in the activation of naive B cells that recognize these S protein epitopes at first and then induce mechanisms of affinity maturation and memory generation [8]. Therefore, mRNA vaccination induces a B cell memory response [9]. mRNA vaccines effectively prevent severe disease, but they do not prevent infection; consequently, vaccinated individuals could be infected by SARS-CoV-2 [10]. In this context, SARS-CoV-2 initiates the immune response in which there are already memory B cells that recognize epitopes of the S protein. Therefore, common epitopes among SARS-CoV-2 variants, new epitopes generated by the variants, and epitopes from other SARS-CoV-2 proteins will be presented in the lymph node. Regarding the common epitopes, a rapid and intense memory response will be initiated, and in our study, we have shown how the antibody titers against S protein after the infection were >34 times higher than the previous titers presented by the HCWs (Table 1). For new epitopes or epitopes from other SARS-CoV-2 proteins, a primary immune response will be developed. Our study analyzed the primary response against N protein, and we have shown that vaccinated HCWs (S protein memory response) developed an antibody response less than half of what nonvaccinated HCWs developed (Table 1). Further studies analyzing the antibody response against new S protein epitopes from SARS-CoV-2 variants in homogeneous groups of vaccinated and nonvaccinated primary infected patients are needed to elucidate whether the reduced response observed against N protein could also occur for S protein and the magnitude of this effect, in which in the case of anti-N protein antibodies, our study showed a reduction of 53.3% of the median response. This phenomenon should be taken into account in the development of vaccines against these new variants.

In response to human coronaviruses, neutralizing antibodies typically bind to the S protein and disrupt viral entry by blocking interactions between viruses and ACE2 host receptors [11]. However, N proteins from different viruses used as vaccines can protect the hosts against the virus infections [11]. Severe infection was associated with enhanced anti-S and anti-N protein antibody responses, and high anti-N protein titers were especially observed in patients who suffered long hospitalizations. [12]. Although the response against the N protein is important for the protection against SARS-CoV-2 infection, the inhibition observed in our research in HCWs who have developed a memory response against the S protein, induced by the vaccine, probably does not have a significant effect on protection against infection due to the great efficacy of the S protein memory response against SARS-CoV-2 [13].

Our study has limitations. The group of HCWs vaccinated with primary infection is low (*n* = 55), but it is enough to achieve statistical significance. The study population was HCW; therefore, our cohort is heterogeneous from a socioeconomic point of view and is representative of the general population restricted to healthy individuals, aged from 18 to 65. The antibody titers against N protein developed by primary infected HCWs could be underestimated since the commercial immunoassay detects antibodies against the original N protein and not the variant N protein. In addition, this commercial immunoassay was semiquantitative (index).

In conclusion, our results showed that the vaccine-induced immune imprinting against the S protein partially inhibits the response against the N protein after SARS-CoV-2 infection.

## 4. Methods

### 4.1. Patients

Two hundred thirty-nine HCWs who had a primary infection by SARS-CoV-2 with a PCR positive test were included. Among these subjects, 51 HCWs were vaccinated with mRNA vaccines against S protein (44 with BTN162b2 and 7 with mRNA-1273) and 188 were nonvaccinated. In the vaccinated group, antibodies against S protein were determined 123 ± 20 days after the second dose of mRNA vaccine, and all primary infections occurred after vaccination. Age, sex, antibodies against S and N proteins before and after infection, the time interval between infection, and/or vaccination and serology were collected from the medical history database (Table 1). The study was approved by the Drug Research Ethics Committee of Parc Taulí University Hospital, Sabadell, under reference 2021/5112.

### 4.2. Detection of SARS-CoV-2 Antibodies

Antibody response to SARS-CoV-2 S protein was measured using the Elecsys® Anti-SARS-CoV-2 S test (quantitative), while antibody response to SARS-CoV-2 N protein was measured using the Elecsys® Anti-SARS-CoV-2 IgM/IgA/IgG test (Roche Diagnostics International Ltd., Rotkreuz, Switzerland, semiquantitative) according to the manufacturer's instructions. Lack of response to the N protein was defined at anti-N protein antibody titers lower than one. The titers of anti-S protein antibodies have an upper limit of 25,000 BAU/mL since the last dilution performed in the routine analysis is 1/100; samples with titers higher than 25,000 BAU/mL have been assigned 25,000 BAU/mL.

### 4.3. Statistical Analysis

For descriptive purposes, the cohort was characterized using absolute and relative frequencies for categorical variables, while medians were used for numerical measures. We tested the association between variables with the chi-square for categorical variables and the Mann-Whitney U test or Student's *t*-test for continuous quantitative variables. The quantitative differences in anti-protein N antibodies between groups were evaluated using linear regression models, analyzing the coefficient variation after introducing the different variables in these models. The variation of the coefficient was <5%. Therefore, a univariate analysis was applied using the Mann-Whitney *U* test, given the nonparametric nature of the antibody response against N protein.

## Figures and Tables

**Table 1:** Demographic and analytical characteristics of HCWs included in the study.

	HCWs vaccinated (*n* = 55)	HCW nonvaccinated (*n* = 188)	*p* value
Sex (women)	80.4%	75.0%	0.423
Age (years)	40.9 ± 11.5	41.3 ± 12.7	0.809
Time interval infection to serology (days)	22.0 (IQR 14.0-45.0)	18.0 (IQR 11.0-34.0)	0.379
S protein antibodies before infection (BAU/mL)	703.0 (IQR 383.0-1362.0)	N.D.	—
N protein antibodies before infection (index)	0.09 (IQR 0.09-0.10)	0.09 (IQR 0.08-0.10)	0.953
S protein antibodies after infection (BAU/mL)	24,062.0 (IQR 11,753.0-25,000.0)	N.D.	—
N protein antibodies after infection (index)	5.7 (IQR 2.3-15.2)	12.2 (IQR 4.2-32.0)	0.005

Categorical variables are expressed in frequencies, and continuous variables are expressed in median and interquartile range for nonparametrical variables and in mean and standard deviation for parametrical variables. *p* values were obtained using the chi-square test for categorical variables and the Mann-Whitney *U* test (nonparametrical) or Student's *t*-test (parametrical) for continuous quantitative variables. HCWs: healthcare workers; IQR: interquartile range; S protein: spike protein; N protein: nucleocapsid protein BAU: binding antibody units; N.D.: not determined.

## Data Availability

The data that support the findings of this study are available from the corresponding author upon reasonable request.
